# Rapid Detection of high-level oncogene amplifications in ultrasonic surgical aspirations of brain tumors

**DOI:** 10.1186/1746-1596-7-66

**Published:** 2012-06-12

**Authors:** Long N Truong, Shashikant Patil, Sherry S Martin, Jay F LeBlanc, Anil Nanda, Mary L Nordberg, Marie E Beckner

**Affiliations:** 1 Department of Biological Sciences, Louisiana State University - Shreveport, One University Place, Shreveport, LA 71115, USA; 2 Department of Neurosurgery, Baylor College of Medicine, 1327 Lake Point Pkwy, Suite 400, Sugar Land, TX 77478, USA; 3 Delta Pathology Group, One Saint Mary Place, Shreveport, LA 71101, USA; 4 Department of Emergency Medicine, Long Medical Center, Louisiana State University Health Sciences Center – New Orleans, 5825 Airline Hwy, Baton Rouge, LA 70805, USA; 5 Department of Neurosurgery, Louisiana State University Health Sciences Center – Shreveport, Rm. 3-215, 1501 Kings Highway, Shreveport, LA 71130, USA; 6 Feist-Weiller Cancer Center, Louisiana State University Health Sciences Center – Shreveport, Rm. B-215, 1501 Kings Highway, Shreveport, LA 71130, USA; 7 Departments of Pediatrics & Medicine, Louisiana State University Health Sciences Center-Shreveport, Rm. 2-303, 1501 Kings Highway, Shreveport, LA 71130, USA; 8 Delta Pathology Molecular Diagnostics, One Saint Mary Place, Shreveport, LA 71101, USA; 9 Department of Neurology, Louisiana State University Health Sciences Center – Shreveport, Rm. 3-438, 1501 Kings Highway, Shreveport, LA 71130, USA

## Abstract

**Background:**

Genomic tumor information, such as identification of amplified oncogenes, can be used to plan treatment. The two sources of a brain tumor that are commonly available include formalin-fixed, paraffin-embedded (FFPE) sections from the small diagnostic biopsy and the ultrasonic surgical aspiration that contains the bulk of the tumor. In research centers, frozen tissue of a brain tumor may also be available. This study compared ultrasonic surgical aspiration and FFPE specimens from the same brain tumors for retrieval of DNA and molecular assessment of amplified oncogenes.

**Methods:**

Surgical aspirations were centrifuged to separate erythrocytes from the tumor cells that predominantly formed large, overlying buffy coats. These were sampled to harvest nuclear pellets for DNA purification. Four glioblastomas, 2 lung carcinoma metastases, and an ependymoma were tested. An inexpensive PCR technique, multiplex ligation-dependent probe amplification (MLPA), quantified 79 oncogenes using 3 kits. Copy number (CN) results were normalized to DNA from non-neoplastic brain (NB) in calculated ratios, [tumor DNA]/[NB DNA]. Bland-Altman and Spearman rank correlative comparisons were determined. Regression analysis identified outliers.

**Results:**

Purification of DNA from ultrasonic surgical aspirations was rapid (<3 days) versus FFPE (weeks) and yielded greater amounts in 6 of 7 tumors. Gene amplifications up to 15-fold corresponded closely between ultrasonic aspiration and FFPE assays in Bland-Altman analysis. Correlation coefficients ranged from 0.71 to 0.99 using 3 kit assays per tumor. Although normalized CN ratios greater than 2.0 were more numerous in FFPE specimens, some were found only in the ultrasonic surgical aspirations, consistent with tumor heterogeneity. Additionally, CN ratios revealed 9 high-level (≥ 6.0) gene amplifications in FFPE of which 8 were also detected in the ultrasonic aspirations at increased levels. The ultrasonic aspiration levels of these amplified genes were also greater than 6.0 CN ratio, except in one case (3.53 CN ratio). Ten of 17 mid-level (≥3.0 & <6.0 CN ratio) amplifications detected in FFPE were also detected as being increased (≥ 2.0 CN ratio) in the aspirations.

**Conclusions:**

Buffy coats of centrifuged ultrasonic aspirations contained abundant tumor cells whose DNA permitted rapid, multiplex detection of high-level oncogene amplifications that were confirmed in FFPE.

**Virtual slides:**

http://www.diagnosticpathology.diagnomx.eu/vs/1883718801686466

## Background

Oncogenes encode proteins that promote tumor growth, survival under adverse conditions, and invasion. Many studies have detected amplified oncogenes in high grade brain tumors, especially glioblastoma or glioblastoma multiforme (GBM). Therefore, assays to identify amplified genes are proposed to become critical in patient care as monoclonal antibodies and small molecules that inhibit proteins encoded by oncogenes become available. Identification of amplified genes in a rapid, multiplex manner is relevant for stratifying patients to clinical trials and treatments. Brain tumor specimens for molecular studies include ultrasonic surgical aspirations available at the time of surgery and small, diagnostic biopsies that are processed as formalin-fixed, paraffin-embedded (FFPE) samples. Sometimes tumor fragments are resected and are also processed as FFPE samples. Although tissue frozen for storage at the time of surgery can also be released for DNA studies, it is not usually available outside of research settings. Ultrasonic surgical aspirations are not routinely used for diagnostic purposes and represent an untapped source of abundant, fresh tumor DNA. With laboratory support for processing cellular fluids and the expertise for confirming tumor cell content morphologically, pathology departments are well suited for providing cellular or nuclear pellets from ultrasonic surgical aspirations to provide tumor DNA needed in molecular testing. Surgical aspirations can quickly provide DNA to speed up turnaround times and produce higher yields than FFPE sections of small biopsies.

Brain tumors debulked by ultrasonic surgical aspiration yield suspensions of single cells, minute tissue fragments, and blood in saline. Following introduction of this technique in the 1970’s, ultrasonic aspiration became routine in microneurosurgery [1]. Unlike the characteristically small diagnostic biopsies of brain tumors, ultrasonic surgical aspirations contain ample tumor cells in large volumes of fluid. The ultrasonic apparatus consists of a computerized control unit with settings for different amplitudes of ultrasonic waves and speeds of irrigation and aspiration. Tumor tissues are targeted and selectively aspirated. An advantage of the ultrasonic aspiration technique over tissue dissection is the reduced need for retraction of normal brain in the patient. Accordingly, the use of ultrasonic surgical aspiration to debulk intracranial tumors is common and explains the unfortunate paradox of having only small diagnostic tissue biopsies from large brain tumors available for molecular studies outside of research settings. Although ultrasonic aspiration specimens have yielded viable tumor cells for experimental studies [2-4], their lack of tissue architecture greatly diminishes their usefulness in diagnostic pathology. For example, regions of tumor necrosis that are a useful diagnostic feature in high grade tumors are prone to disintegrate during the aspiration procedure. Although some institutions preserve small portions of ultrasonic aspirations in FFPE blocks, the intact tissue fragments from biopsies are preferred and routinely relied upon for diagnostic evaluation of primary and metastatic brain tumors. Ultrasonic surgical aspirations, that contain the bulk of brain tumor tissue in a dispersed and disaggregated form, seldom provide the complete architectural features of the tumors that aid in determining morphologic diagnoses.

In this study, ultrasonic aspirations of brain tumors were tested to see if they would yield tumor DNA that is appropriate for studies to detect amplified oncogenes. Testing methods that identify amplified genes as potential treatment targets in individual tumors would ideally be performed as soon after surgery as possible. The genes tested with commercial MLPA kits are known to be amplified in association with malignancy. The amounts of DNA purified from ultrasonic aspirations of GBMs were found to be especially ample. These DNA samples were analyzed for amplifications in seventy-nine oncogenes, with results compared to those from FFPE-derived DNA and with fluorescence *in situ* hybridizaiton (FISH) results for *EGFR* gene amplification in FFPE tissue sections. The MLPA assays on ultrasonic aspirations identified high-level amplified genes within a few days at low cost and served as a preview of the more sensitive FFPE assay results that became available later.

## Methods

### Patient Population, Specimen Retrieval and DNA Purification

Permission from our Institutional Review Board was obtained for retrieval and study of the excess amounts of ultrasonic aspiration and FFPE specimens taken from brain tumors resected in 2008–2009 in a pilot study of using these specimens for molecular tests. Ultrasonic aspirations from seven brain tumors, including four glioblastomas (GBM1-4), two lung carcinomas metastases (LCM1-2) and an ependymoma (EP1), were tested. The patients (all male except for one female with a GBM) were 44 to 81 yrs of age. Samples were obtained in saline with a Cavitron Ultrasonic Aspirator Excel (Valleylab-Tyco International, Boulder, CO) set at 23 to 36 MHz. The volume (cc) of each tumor present preoperatively and postoperatively was estimated by the neurosurgeon. The ultrasonic surgical aspirations were not designated to be used for any diagnostic purpose. The specimens in this study were delivered to the laboratory within 24 hr of surgical resection, either fresh or after overnight refrigeration. Portions of ultrasonic aspiration specimens, 15 to 30 ml, were centrifuged at 500 g for 10 min. Tumor cells sedimented as large buffy coats above the erythrocytes and beneath floating necrotic debris. According to the manufacturer’s recommendations (Qiagen Midi Kits, Valencia, CA), 500 μl of each buffy coat were pipetted and combined with purification kit components to obtain a nuclear pellet that was processed immediately or frozen for later purification. All specimens yielded measureable amounts of DNA. For comparisons in each tumor, DNA from routine FFPE sections (10 μm thick, 10 per tumor) was purified with a Qiagen FFPE DNA purification kit. In each case tumor cells represented at least 90% of nucleated cells in the tissue sections. Proteinase K digestions at 55°C were extended as needed to achieve digestion of the tissues. Purified DNA was evaluated and quantified with spectrometry using absorbance measurements at 260 and 280 nm (JENWAY Genova, Jenway Limited, Essex, England).

### Histological Review of Tumor Specimens

Diagnostic FFPE tissue sections, available from all tumors, were reviewed microscopically to characterize the pathological features. Portions of ultrasonic aspiration specimens that had also been processed as FFPE specimens were available. Hematoxylin and eosin stained FFPE sections were evaluated. The number of mitotic figures in ten high power fields (HPFs) (400X magnification) was determined for each tumor in duplicate counts. Also, stains for Ki67 reactive cells were performed at the time of diagnosis for some tumors using a pre-diluted antiKi67 antibody (Ventana Medical Systems, Oro Valley, AZ) for direct staining on an automated immunohistochemical stainer (Benchmark XT, Ventana Medical Systems). Pre-diluted, non-reactive mouse monoclonal negative control (Ventana) solutions were applied to separate tissue sections. The percentages of tumor cells positive for Ki67 specific staining were determined with an automated cellular imaging system (Chromavision ACIS, San Juan Capistrano, CA) with verification by manual counts at the time of review. If interference from background staining was present, manual counts were substituted.

### *EGFR* FISH

Inclusion of probe sets for *EGFR* in the MLPA kits permitted comparison of MLPA and FISH copy number (CN) data for this gene in each tumor. FISH probes for a control locus, 7p11.1-q11.1, D7Z1, and the *EGFR* band region, 7p11.2-7p12, (Vysis Locus Specific Identifier (LSI) *EGFR* SpectrumOrange/CEP 7 SpectrumGreen, Abbott Molecular Inc., Des Plaines, IL) were hybridized to interphase nuclei. Paraffin sections of all tumors and cytologic smears prepared from some of the ultrasonic aspiration specimens were examined. Smears from aspirations were fixed, denatured, and hybridized with probes overnight. Un-hybridized probes were washed away. Diamino-phenylindole (DAP1) fluorescent blue (Abbott Molecular) stained the nuclei. Slides were scanned on a fluorescent microscope (Leica DMR, Wetzlar, GM) for analysis with images captured using a digital camera (Applied Imaging, San Jose, CA) and CytoVision v4.02 imaging software (Applied Imaging). Sections from FFPE tissue were cut at a thickness of five microns, deparaffinized (Paraffin Pretreatment Reagent Kit II, Abbott Molecular), processed on a VP2000 Processor (Abbott Molecular) and then FISH probes for *EGFR* and CEP 7 were applied and analyzed as described above. Amplifications of *EGFR* were observed as average *EGFR* to CEP 7 signal ratios greater than two for at least 20 cells (usually many more). The results were determined by averages of all cells that could be evaluated. Proteinaceous debris hindered FISH interpretations in some of the ultrasonic surgical aspiration specimens. Ratios of total *EGFR* and CEP 7 signals per tumor, individual *EGFR*/CEP 7 signals per cell and medians of the signal ratios per cell were described.

### MLPA

The PCR-based technique, MLPA, involved multiple steps, including exposure of DNA to gene-specific probe sets, enzymatic ligation, PCR, and capillary electrophoresis (CE) to separate PCR products. Briefly, as explained earlier [5], 200 μg of DNA from each tumor sample and normal brain in buffer were denatured at 98°C for 30 min in a thermocycler (MasterCycler personal Eppendorf, Hamburg, GM) and were then hybridized to MLPA probes (SALSA P171, P172 and P173 kits, MRC-Holland, The Netherlands) according to kit instructions. The manufacturer selected genes according to literature documenting amplification in tumors. In addition to gene specific sequences, the probes also contained universal PCR primers, X and Y, and stuffer sequences for subsequent identification of specific gene amplicons by size using CE. Ligase65 (MRC-Holland) generated ligations specific for perfectly hybridized probe pairs (sets) at ligation junctions in the DNA and the enzyme was then heat inactivated. Ligated probes were PCR amplified with polymerase (MRC-Holland) according to instructions. PCR products were separated on a CEQ 8000 (Beckman-Coulter, Fullerton, CA) and were then identified according to the length of amplicons.

Fragment analysis of PCR products produced a series of linear peaks (signals of fluorescence) corresponding to the relative quantities of PCR products that were proportional to initial amounts of DNA (or CN) of the targeted genes. Slightly less efficient amplifications of longer amplicons accounted for minor reductions in their peak heights. All of the genes tested in the three MLPA kits, P171, P172, and P173, are listed as follows: *AKT1, AURKA, BCAR2, BCAS1-2, BCL2, BCL2A1, BCL2L1*, *11* and *13, BCL6, BCLG, BIRC1-5, BRAF, BRMS1, CCNA1, CCND1-2, CCNE1, CDK4* and *6, CENPF, CYP27B1, EGFR, EMS1, ERBB2* and *4, ESR1, EVI1, FGF3* and *4, FGFR1, FLJ20517, GNAS, GSTP1, HMGA1, IGF1R, IGFBP2,4* and *5, IRS2, JAK2, MDM2* and *4, MET, MMP7, MOS, MYCL1, MYBL1* and *2, MYC, MYCN, NFKBIE, NRAS, NTRK1-3, PDGFRA* and *B, PIK3C2B, PIK3CA, PPM1D, PSMB4, PTK2, PTP4A3, PTPN1, RELA, RNF139, RUNX1, SERBINB2, 7* and *9, TERT* and *TOM1L2*.

Occasional failures to see peaks in one of the eight capillary tubes during a CE run were attributed to low current whenever the peak was obtained after repeating CE. Successful concurrent results for NB were required for a CE run of tumor samples to be further analyzed.

### Normalization of CN

For detection of somatic changes found in highly malignant tissues, there were no known reference probes that could be relied on to maintain their normal CN at the time of this study. Large numbers of genes are lost or amplified in malignant tumors leading to considerable variability in the CN of individual tumors. Also, genetic variations and mutations in tumors, such as nucleotide deletions, insertions, or substitutions near the ligation junctions could potentially impact ligation efficiency. In this study the result of each probe set in each tumor specimen was normalized with CN for the same probe set in DNA from non-neoplastic brain (NB) assayed concurrently. Comparisons of all tumors with the same source of NB controlled for assay to assay variation. Aliquots of NB were from an 82 year old woman’s normal occipital lobe (Biochain, Hayward, CA).

**Figure 1:**
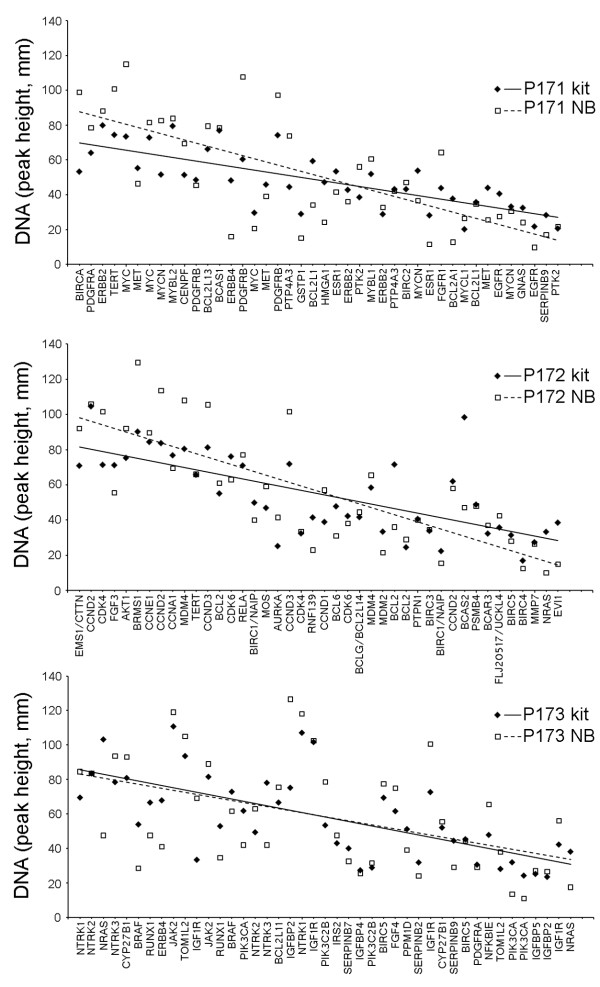
**Scatterplots of DNA values for genes in non-neoplastic brain versus normal values provided in MLPA kits.** The amounts of DNA represent copy number (CN) for each gene assayed. The non-neoplastic (or normal) brain DNA (NB) was evaluated by comparing its values with expected values provided by the manufacturer. Regression analysis of NB values, that were obtained when FFPE-GBM1 was tested with the P171 kit, identified *ERBB4* at the lower limit of the 95 % confidence interval as an outlier. The overlays of observed NB values with normal values included in the P171, P172, and P173 kits demonstrated overall correspondence for both sources of non-neoplastic DNA in the data distributions. Their trendlines are also shown.

The CN ratios, or fold-differences from normal for each gene, are represented by the ratio, [tumor DNA] / [NB DNA], derived from measurements of the CE peaks. The CN ratio was calculated after peaks of non-amplified genes on CE graphs, representing relative amounts of DNA, were matched with the average of two NB samples analyzed in the same assay run. Peak heights of non-amplified genes in tumor samples and NB were adjusted to approximately the same scale as graphical printouts from CE were produced. Final, finer adjustments were made in spreadsheets by maximizing alignments of trendlines for non-amplified genes. Scatterplots were evaluated to check the reactions of NB DNA with the MLPA probes compared to expected values provided by the manufacturer. Overlays of NB results obtained in GBM1’s assays of FFPE, using P171, P172, and P173 kits, with graphs of normal values provided in the MLPA kits’ literature, demonstrated comparable overall detection of the two populations of normal DNA data points (Figure 1).

Regression analysis of NB’s scatterplots demonstrated that results obtained using one of the two probe sets for *ERBB4* was an outlier. In P171 data points, *ERBB4*’s probe set result was at the 95% confidence limit for the overall NB sample population. All other probe set results fell above the lower 95% confidence limit for the NB sample. Also, three probe sets were slightly out of range in the opposite direction so that missing a low level amplification was a concern but other probe sets for these genes were within the 95% confidence limits so that missing a significant CN gain after their results were averaged would be unlikely. The probe sets with results above the confidence limits included 1 of 2 for *IGFBP2*, 1 of 2 for *NTRK1*, and 1 of 4 for *IGF1R*.

The normal CN range was set at ≥ 0.75 to ≤ 1.50, with CN ratios ≥ 2.0 considered to be amplified. High-level gene amplifications in glioblastomas have been previously set at 6-fold greater than diploid or 12 or more copies per nucleus [6]. In this study CN ratios ≥ 6.0 were also designated as high-level amplifications and lesser amplifications were set at CN ratios of ≥ 3.0 and < 6.0 for mid-level and ≥ 2.0 and < 3.0 for low-level amplifications. Averaged results for two replicates of amplified and non-amplified genes in ultrasonic aspiration tumor DNA were compared to results for two replicates of genes in NB for each gene’s probe set (or ligated pair). Results for multiple probe sets for a gene were averaged. MLPA analysis using the kits, P171, P172, and P173, was performed on FFPE DNA in two replicates from each of 7 tumors in 15 of the 21 assays and in single replicates in 6 due to DNA depletion from the small specimens.

### Statistical methods and correction factors

Excel was used to prepare graphs for images, determine Spearman rank correlation coefficients, regression analysis, etc. Outlier identification was performed with regression analysis to detect data points at or beyond 95% confidence intervals for residuals. A Bland-Altman plot to compare MLPA results of cavitronic ultrasonic surgical aspiration and FFPE assays was generated with R, version 2.10 (The R Foundation for Statistical Computing, http//http://www.R-project.org), statistical software.

## Results

### DNA purification yields

Successful DNA purification was achieved for all of the tumors. The greatest yields of DNA (43.0 to 77 μg) were obtained from buffy coats of glioblastoma ultrasonic aspiration specimens. The FFPE sections from all of the tumors yielded less DNA (6.4 to 20.5 μg) as seen in Table 1.

**Table 1:** DNA data according to sample type, cavitronic ultrasonic surgical aspiration (CUSA) or formalin-fixed, paraffin-embedded (FFPE) sections

**Brain tumors**	**Specimen type**	**260/280 Absorbance (nm)**	**DNA yield per specimen (μg)**
GBM1	CUSA	1.38	49.1
	FFPE	1.98	6.7
GBM2	CUSA	1.84	77.0
	FFPE	1.98	7.9
GBM3	CUSA	1.84	43.3
	FFPE	1.86	6.4
GBM4	CUSA	1.67	44.6
	FFPE	1.90	7.0
LCM1	CUSA	1.85	17.9
	FFPE	1.81	11.3
LCM2	CUSA	1.16	14.6
	FFPE	1.63	20.5
EP1	CUSA	1.88	12.1
	FFPE	1.55	10.1

GBM = glioblastoma, LCM = Lung carcinoma metastasis, EP1 = Ependymoma.

### Clinicopathologic features of the tumors

**Figure 2:**
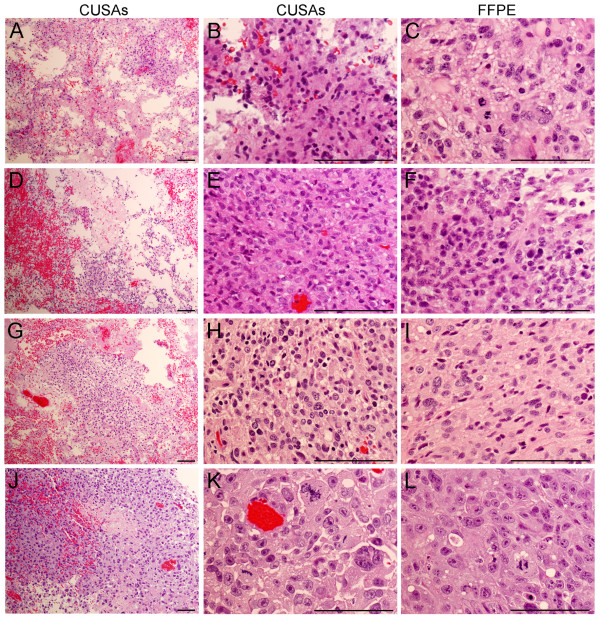
**Morphology of the cavitronic ultrasonic surgical aspiration (CUSA) and FFPE specimens from several tumors.** Specimens for GBM1, GBM2, GBM4, and LCM1 are shown in the first, second, third, and fourth rows, respectively. The original magnification for the first column (panels A, D, G, and J) was at 100X and the others were at 400X. Comparable morphology of the ultrasonic aspiration and FFPE specimens from each tumor is noted in the high power views shown in the last 2 columns. Cellular pleomorphism and mitoses were prominent. Characteristic regions of necrosis and vascular proliferation were also found in the FFPE sections of the GBMs (not shown). Several clinicopathologic features are listed in Table 2. The portions of the CUSA specimens shown had been processed in the same manner as the FFPE specimens. Hematoxylin and eosin stain. Magnifications bars represent 100 micrometers.

Features of the tumors were typical for their diagnostic classification as glioblastomas, metastatic tumors, and an ependymoma. Photomicrographs of the ultrasonic surgical aspirations (100X and 400X magnifications) and FFPE sections (400X magnification) illustrates the similarity of tumor cells in the two types of specimens (Figure 2). There were too few cases to evaluate whether characteristic morphologic profiles for amplified gene(s) in these tumors were present. Mitotic figures among the glioblastomas were most numerous in GBM1 and mitoses among all tumors were most numerous in LCM2 (Table 2).

**Table 2:** Selected clinicopathologic features of the brain tumors in this study

**Tumor**	**Age & sex**	**Tumor (cc)**		**Mitoses per 10 HPF**	**Ki67 positive tumor cells**
		Pre Op	Post Op		
GBM1	50 yr M	80.8	19.1	46.5 ± 6.4	61 %
GBM2	81 yr M	51.4	0.0	19.5 ± 2.1	55 %
GBM3	52 yr F	124.0	96.1	10 ± 2.8	38 %
GBM4	44 yr M	35.6	0.0	18.5 ± 2.1	Not tested
LCM1	55 yr M	19.0	0.0	51.5 ± 9.2	Not tested
LCM2	61 yr M	6.5	0.0	64.5 ± 5.0	Not tested
EP1	73 yr M	20.6	1.38	0	10 %

The volumes of tumor were provided as estimates by the neurosurgeon. HPF = high power field (400X).

### *EGFR* FISH

**Figure 3:**
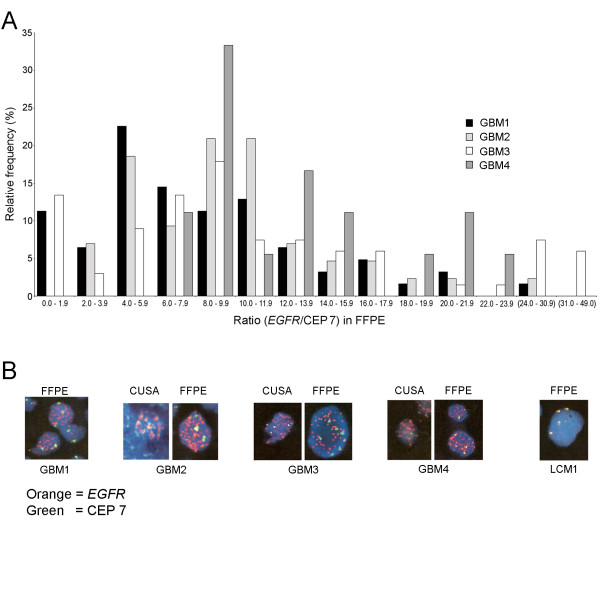
**FISH results for *EGFR* are shown.** A. Relative frequencies of *EGFR*/CEP 7 signals (number of cells in intervals/total cells counted multiplied by 100) in FFPE sections of glioblastomas are shown on the *y*-axis in distributions of signal ratios. Data is skewed to the right due to cells that harboured very high numbers of *EGFR* signals in each tumor, especially GBM3 and GBM4. Intervals of values for signal ratios along the *x*-axis are equal except for the last two which are arbitrarily larger (in parentheses). B. Representative tumor cells show *EGFR* (orange/red) and CEP 7 (green) signals. Nuclei were stained blue with DAPI. All tumors had FFPE sections analyzed and a few also had CUSA specimens analyzed successfully. Others had interference from proteinaceous debris. High power photographs were printed in large format and then digitized.

Analysis of *EGFR* with FISH was used to correlate with MLPA assay results. Signals were counted in the FFPE sections. The CN for *EGFR* was increased in the four glioblastomas with ratios of *EGFR* to CEP 7 increased to values of more than 20 in at least some cells of each glioblastoma. In the metastatic tumors averages of CNs for *EGFR* were 2.5 to 2.8 per cell with *EGFR* to CEP 7 ratios remaining below 2.0. Distributions of the *EGFR*/CEP 7 ratios are shown in Figure 3A and illustrate “tailing off” in the data towards high-level amplifications in individual cells. Representative cells with amplified *EGFR* from the glioblastomas and from one of the metastases lacking amplification are shown in Figure 3B. The EGFR/CEP 7 ratios are listed for all tumors in Table 3. The CN of CEP 7 varied and was increased (averages of 2.7 to 3.8) in glioblastomas. The CN of CEP 7 varied in LCM1 and LCM2 with averages of 2.5 and 2.6, respectively.

**Table 3:** **FISH versus MLPA results for *EGFR* **

**Tumor**	***EGFR*/CEP 7 ratios in FISH assays on FFPE**		***EGFR*’s tumor/NB DNA ratios (normalized CN) Average of 2 MLPA probe sets ± SD**	
	Total *EGFR/*Total CEP 7 signals	Median ratio	CUSA	FFPE
GBM1	7.3	7.2	8.8 ± 0.2	21.1 ± 2.0
GBM2	8.6	9.3	22.5 ± 7.9	19.3 ± 7.2
GBM3	10.3	9.0	10.5 ± 3.8	33.1 ± 17.2
GBM4	11.1	11.6	19.5 ± 9.3	26.7 ± 6.5
LCM1	1.0	1.0	1.2 ± 0.5	1.2 ± 0.4
LCM2	1.1	1.0	2.3 ± 0.9	2.3 ± 1.4
EP1	1.0	1.0	1.7 ± 0.7	2.9 ± 2.6

Although amplification of *EGFR* seen in individual cells of GBMs varied greatly and MLPA provided collective results of numerous cells, both techniques demonstrated corresponding large elevations in copy number for this oncogene in GBMs. Polysomy may have accounted for low-level amplifications found with MLPA in LCM2 and EP1. Median ratios were derived from the distributions of signal ratios in individual cells. *EGFR* = *Epidermal Growth Factor Receptor*. CEP 7 = Centromere of chromosome 7, FISH = Fluorescence *in situ* hybridization. SD = Standard deviation. CUSA = Cavitronic ultrasonic surgical aspiration. FFPE = Formalin-fixed, paraffin embedded tissue.

### MLPA molecular results

**Figure 4:**
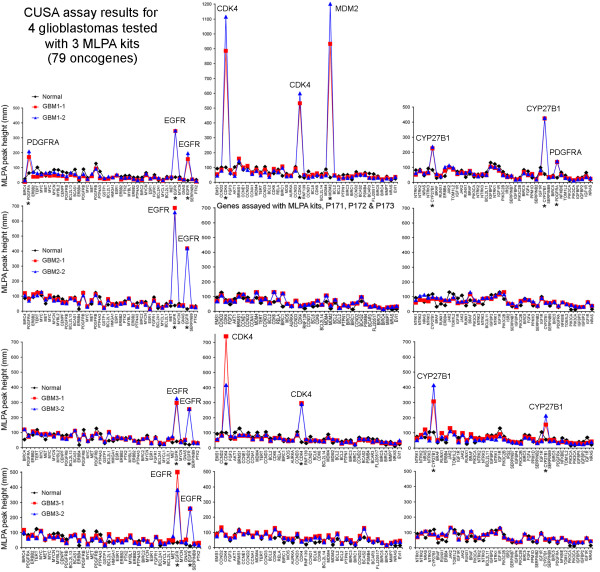
**Multiple gene amplifications detected with MLPA in GBM1-4 shown as line graphs of peak heights.** MLPA results for genes in tumors were compared with non-neoplastic (or normal) brain DNA. Heights of peaks reflected amounts of genes, or their CN. Mid to high-level gene amplifications were obvious by visual inspection of peak heights (mm) for at least one gene in each glioblastoma. These are labelled and also asterisks indicate these genes along the *x*-axes. Line graphs connecting the peak heights are colored according to the legends. Tumor and normal brain (NB) were tested concurrently.

Productive CE runs were obtained for all tumors. Prior to normalization of tumor CN, the output of CE data from all four glioblastomas and one of two metastases revealed obvious amplifications in at least one gene for each tumor. Measurements of the peaks for each gene were connected to create line graphs. The obvious peaks representing high-level and some mid-level gene amplifications in the glioblastomas are shown in Figure 4. Genes are listed along the *x-*axes according to amplicon size within each kit (P171, 172 and 173). Easily recognized amplifications occurred for *EGFR*, *CDK4*, *MDM2*, *CYP27B1*, and *PDGFRA* in one or more GBMs*,* and also for *CCNE1* in one of the brain metastases (not shown) using DNA from ultrasonic surgical aspirations. Comparable amplifications for the same genes were also detected in corresponding FFPE specimens (not shown). Results for non-amplified genes in the tumors and in normal brain (assayed concurrently) constitute the baselines of graphs in Figure 4. The *EGFR* data obtained with MLPA is included in Table 3 along with FISH data.

**Figure 5:**
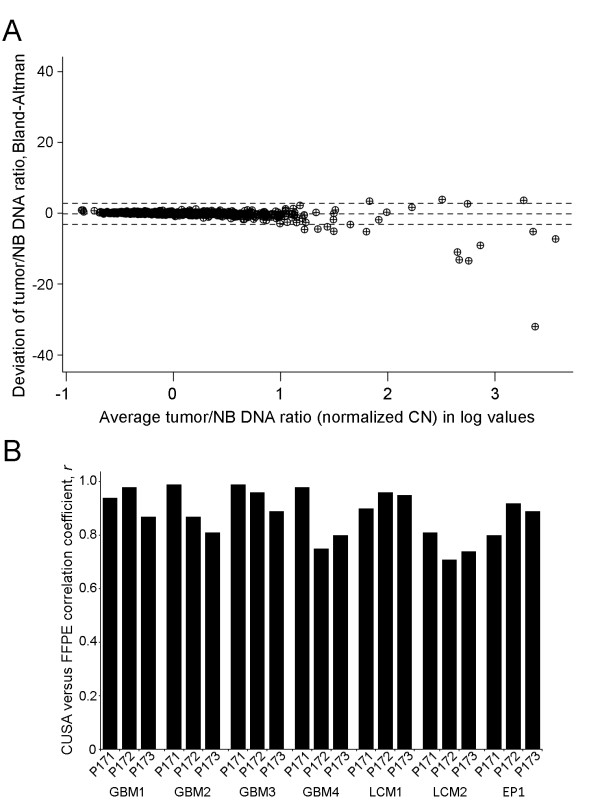
**Comparisons between cavitronic ultrasonic surgical aspiration (CUSA) and FFPE specimens for all normalized CN ratios.** A. Bland-Altman analysis demonstrated that normalized CN ratios ([tumor DNA]/[NB DNA]) obtained from either source of DNA corresponded significantly (within 1.96 SD limits, dotted lines) for oncogene amplifications up to at least 15-fold (log value of 1.18). The logarithmic scale was used on the *x*-axis to separate ratio values for the majority of the data points as much as possible. Note that a normal CN ratio of 1 is equivalent to a log value of 0. The CN ratios for all genes tested with each kit (multiple probes) in all tumors were included, *n* = 889. B. The CN ratios derived from the two sources of DNA exhibited strong correlations in each tumor with results from each kit shown separately. Assays using the MLPA kits, P171, P172, and P173, had *n*’s equal to 42, 42 and 43, respectively.

Following NB normalization to generate CN ratios, Bland-Altman analysis (*n* = 889, derived from 127 data points/tumor x 7) found that data in ultrasonic surgical aspiration assays corresponded to data from FFPE assays for the same tumor within 1.96 SD limits, except for some of the amplifications that exceeded CN ratios of 15 (log value = 1.176). A normal CN ratio of 1 corresponds to a log value of 0 (Figure 5A). Correlation coefficients for comparisons ranged from 0.71 to 0.99 in studies of each tumor according to the kits used. Consistency was high with the exception of LCM2 that showed slightly less correspondence, as seen in Figure 5. The *n*’s were 42, 42, and 43 for P171, P172, and P173 kits, respectively, in each tumor.

**Figure 6:**
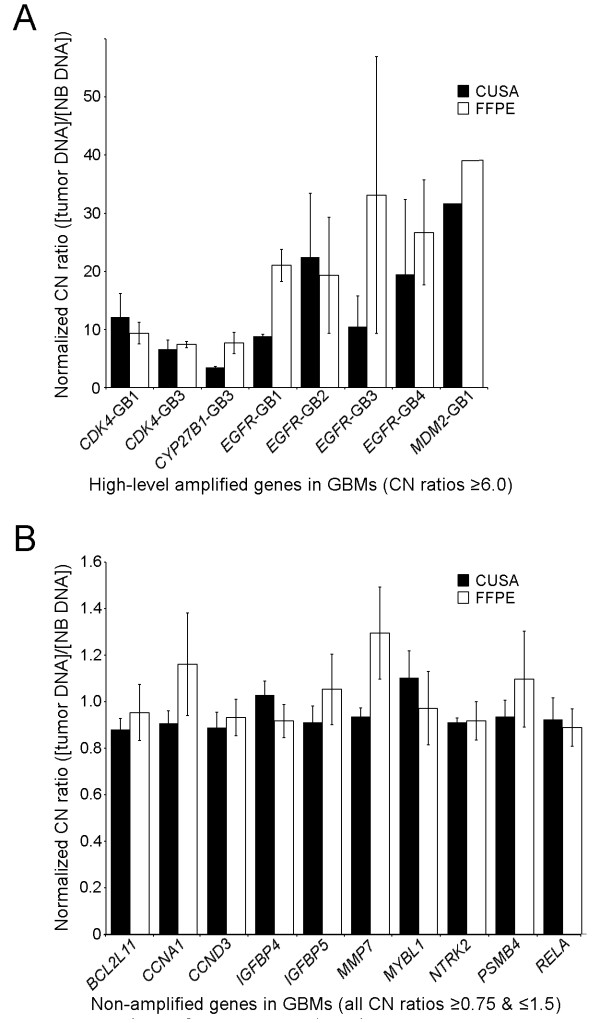
**Individual CN ratios of selected genes in CUSA and FFPE specimens from all four glioblastomas.** A. The CN ratio values for genes with high-level amplifications (CN ratios ≥ 6.0) are shown for individual glioblastomas as indicated along the *x-*axis. Each gene, except for *MDM2*, was tested with two probe sets. B. In contrast, the CN ratio values are shown for individual genes that were unaltered within all four glioblastomas. These CN ratios remained in the normal range for all replicates and probe sets in all assays on both types of glioblastoma specimens. Note the marked difference between the ranges of *y*-axes for A and B. The CN ratios were calculated according to [tumor DNA] / [NB DNA] with averages in 95 % confidence intervals (bars) shown here.

Normalized CN ratios for specific genes in glioblastomas, including those with high-level amplifications (CN ratio ≥ 6.0) and those with no alterations (all CN ratios ≥0.75 and ≤1.5), are shown in Figure 6 (A & B, respectively). Both the ultrasonic surgical aspiration and corresponding FFPE results from the same tumors are shown. The only high-level gene amplification in the brain metastases was *EVI1* (7.02 CN ratio) in LCM2 FFPE but it was not amplified in the corresponding aspiration specimen. The metastases, LCM1 and LCM2, had 2 and 6 mid-level gene amplifications, respectively. The ependymoma (EP1) only had 1 mid-level gene amplification. Among the 10 genes with no alterations in any of the GBMs, including replicates of all probe sets for a specific gene, there were 9 genes that were also unaltered in EP1, and 9 and 6 genes that were unaltered in LCM1 and LCM2, respectively. Totals of all deviations (<0.75 or ≥2.0 in CN ratios) from normal values in ultrasonic aspiration and FFPE specimens are shown in Table 4. Alterations in CN were more frequent in FFPE than in ultrasonic aspiration specimens, 85% and 49% of total changes, respectively. However, FFPE did not completely encompass all of the changes. Restriction of some genomic gains and losses to only one type of specimen from the same tumor is consistent with tumor heterogeneity.

**Table 4:** Copy number changes in formalin-fixed, paraffin-embedded (FFPE) and cavitronic ultrasonic surgical aspiration (CUSA) specimen DNA

**Tumors**	**Genes with normalized CN ratios ≥ 2.0 (gains in copy number)**					**Genes with normalized CN ratios < 0.75 (losses in copy number)**				
	Total	FFPE	CUSA	Only FFPE	Only CUSA	Total	FFPE	CUSA	Only FFPE	Only CUSA
GBM1	18	17	7	11	1	16	12	6	10	4
GBM2	10	10	5	5	0	21	17	9	12	4
GBM3	13	13	7	6	0	21	18	8	13	3
GBM4	10	8	8	2	2	15	13	7	8	2
LCM1	7	7	5	2	0	15	12	11	4	3
LCM2	15	13	5	10	2	32	22	18	14	10
EP1	7	7	3	4	0	16	15	6	10	1
Totals	80	75	40	40	5	136	109	65	71	27

GBM1-4 had increased CN ratios for 22 genes (*AURKA, BCL2A1, BCL6, BIRC1,2,4, BRAF, CDK4,6, CYP27B1, EGFR, ERBB4, ESR1, EVI1, GNAS, GSTP1, MDM2, NRAS, PDGFRA, PIK3CA, RNF139,* &*SERPINB9*) and decreased CN ratios for 35 (*AKT1, BCAR3, BCAS1, BCL2, BCL2L13, BCLG, BIRC5, BRMS1, CCND1, CCNE1, CDK4, CENPF, EMS, FGF4, FGFR1, FLJ20517, GSTP1, IGF1R, IGFBP2, IRS2, JAK2, MDM4, MYBL2, MYC, MYCL1, MYCN, NFKBIE, NTRK1, PDGFRB, PIK3C2B, PTK2, PTP4A3, PTPN1, TERT,* &*TOM1L2*). LCM1-2 had increased CN ratios for 16 genes that overlapped with those for GBM1-4 plus *CCNE1, MET, PPM1D,* and *PSMB4*, and decreased CN ratios were found for thirty six genes that overlapped with those listed for GBM1-4 plus *BCL2L1, BIRC3, CYP27B1, MMP7, NTRK2, NTRK3, PDGFRA, RELA,* &*SERPINB2*. EP1 had increased CN ratios for genes among those for GBM1-4 and decreased CN ratios for those for GBM1-4 or LCM1-2 plus *CCND3, HMGA1*, &*MOS.*

## Discussion

### Overview of tumor genomics

Brain tumors are known for harbouring genetic abnormalities. Tumor genomes are being evaluated to varying extents by FISH, array comparative genomic hybridization (aCGH), single nucleotide polymorphism (SNP) arrays, specific mutation detection with various PCR methods, methylation studies, targeted sequencing of selected genes, and whole genome sequencing. Therapies are planned accordingly in some institutions. Ideally, molecular testing for potential treatment targets occurs at the time of diagnosis and again in tumor recurrences to indicate appropriate treatment alterations.

### Amplified oncogenes in tumor fluids

This study demonstrates that high-level amplified oncogenes can be quickly detected by inexpensive multiplex, PCR-based studies of cellular fluids using standard molecular laboratory equipment and techniques. Fluids with significant tumor cellularity offer the opportunity to retrieve fresh tumor DNA in abundant amounts. Advantages in testing the DNA of tumor cells in fluids rather than FFPE tissue sections, include avoidance of exposure to formalin, heat, and organic solvents, and enrichment for tumor via pelleting, filters, and various other methods, such as those developed for isolating tumor cells from blood. Tumor cells in fluids not needed diagnostically constitute as a potential source of DNA for molecular assays. This study demonstrated detection of amplified oncogenes despite concerns regarding ultrasonic, mechanical, and osmotic distortions and mixing of the tumor cells with saline and blood. Endothelial cells were the predominant type of non-tumor cell present but these do not exceed what was originally present in the tumor parenchyma. A tendency for short sections blood vessels to remain intact and sediment differently from tumor cells was noted but was not investigated.

### Adaptive and Co-amplification of Oncogenes

It is proposed that oncogenes can be adaptively amplified in tumor cells to increase key gene products while circumventing promoters and other traditional methods of gene regulation [7,8]. Tumor cells are already well known for developing resistance to chemotherapy by being permissive to amplification of a gene that encodes a key protein that aids drug metabolism [7]. Although genetic instability of brain tumors may account for some genes being amplified by chance, amplification of multiple oncogenes involved in a similar function, such as cellular proliferation or resistance to apoptosis, suggests an adaptive response. Co-amplification of multiple oncogenes that provoke redundant malignant behavior or adaptation to cancer treatments strengthens the rationale for planning multi-agent therapies based on identification of amplified oncogenes with multiplex techniques.

Although co-amplifications of several oncogenes have been reported in glioblastomas, the lack of predictable patterns in individual tumors indicates that each tumor needs to be tested. Despite a tendency for genes in close proximity to be amplified together in tumor genomes, the span of amplifications in these regions is variable and frequently interrupted. Amplified expanses of chromosomes include both “driver” and “passenger” or “bystander” genes in regard to their effects on tumor behaviour. The benefits of amplified “driver” genes could underlie retention of amplified chromosomal regions in malignant tumor clones.

### Specific oncogenes amplified in glioblastomas

Several oncogenes are commonly amplified in brain tumors. Amplification of *EGFR* or one of its variants, *EGFRvIII,* whose encoded protein is constitutively active, is well-known to occur in primary glioblastomas. Amplification of *EGFR* has also been found in anaplastic astrocytomas [9]. Gains of *EGFR* occurred in 70% of 40 glioblastomas in one study with high levels of gene amplification occurring as double minutes in 42% of the cases. Lower levels of *EGFR* amplification occurred as insertions of extra gene copies distributed along chromosome 7 [10]. In a previous MLPA study of 104 glioblastomas, 74 (71%) had additional copies of *EGFR*[11]. Although high levels of *EGFR* most likely underlie some malignant adaptations in glioblastomas, only small subsets of patients have responded to therapies targeted to EGFR in clinical trials [12-15]. Five moderate to high-level amplified genes in glioblastomas identified in this study, *EGFR, PDGFRA, CDK4, MDM2* and *CYP27B1*, have also been previously reported, sometimes with co-amplification [16-20]. Although proximity of the locations for *CDK4CYP27B1* and *MDM2* at 12q14, 12q13.1-q13.3 and 12q14.3-q15, respectively, contributes to co-amplification, this cannot be assumed to occur for all three genes in individual tumors. In 20 glioblastomas that harbored at least one of these amplifications, only 7 had amplification of all 3 genes [20]. In another study when 5 glioblastomas contained amplification of either *MDM2* or *CDK4*, both genes were amplified in only 3 [18]. In another study, among the 5 glioblastomas that contained amplification of either *CDK4* or *MDM2*, only one tumor had both amplified [17]. In a survey of 456 glioblastomas, 13.4% had amplifications of *CDK4* but only 9.2% had amplification of *MDM2*[16]. In our study, 2 of 4 glioblastomas had co-amplifications of *CDK4* and *CYP27B1* and only one also had amplification of *MDM2.* Amplification of *EGFR* was present in all four glioblastomas.

### Amplifications of oncogenes in other brain tumors

Brain metastases from lung primaries in this study also contained amplified oncogenes suggesting that genomic analysis of metastases will detect amplified genes to serve as treatment targets, such as *CCNE1* that encodes cyclin E1 [21,22]. Additional analyses of brain metastases are indicated to identify the full range of oncogenes that can be amplified. Interestingly, in a previous MLPA study of non-typical meningiomas, 19 oncogenes were found to have amplifications in two or more invasive/atypical/anaplastic (mostly Grade II) meningiomas (total of 15) and the sums of copy numbers were inversely correlated with patient age [5]. Some of those genes were among the high-level amplified genes detected here but none of the amplifications in the non-typical meningiomas were in the high-level range. In this study, sums of CN ratios normalized with NB for the same 19 genes were higher in all 4 glioblastomas and in 1 of the 2 metastases than in the non-typical meningiomas studied previously (not shown).

### Implications of amplified oncogenes

As products of amplified genes become treatment targets, combinations of specific inhibitors and monoclonal antibodies will need to be tailored for individual patients according to amplifications found in the tumor DNA. Additionally, there is a tendency for amplified oncogenes to undergo mutations. Therefore, testing each brain tumor for oncogene amplifications would be useful for detecting key molecular treatment targets.

### Methods for assessments of oncogenes

With multiplex PCR methodology, rapid assessment of a relatively large group of oncogenes can be obtained using routine molecular laboratory techniques and equipment. In comparison to FISH assays, numerous genes can be tested simultaneously with comparisons to multiple non-amplified genes, whereas the number of FISH targets is limited to fewer genes. However, in this study, FISH did offer the advantage of detecting centromere duplication as a surrogate of chromosomal duplication so that low levels of *EGFR* amplification attributed to polysomy could be predicted. In comparison to the specificity of other PCR-based assays, amplification occurred with MLPA only if the probes hybridized to the gene targets in pairs (probe sets) and then underwent enzymatic ligation based on perfect sequence correspondence at their junctions to the tumor DNA. Also, the use of only one-fourth of the reaction volume for PCR in MLPA further reduced the chances of non-specific carryover of PCR amplicons from previous reactions. However, our study did not compare MLPA with other multiplex PCR techniques. Quantitative real-time PCR using multiwell plates is among other rapid molecular techniques to consider for testing multiple oncogenes simultaneously at low cost.

The validity of MLPA has been supported by other techniques. Detection of gene amplification (*ERBB2* or *Her- 2/neu*) with fluorescent and chromogenic *in situ* hybridization has closely correlated with MLPA results [23,24]. Results of *EGFR* amplification with MLPA have been comparable to detection of increased protein and RNA expression using immunohistochemistry and non-MLPA PCR, respectively [10]. In this study, FISH confirmed MLPA results for the presence or absence of mid and high-level *EGFR* amplification. Correlations were not quantified due to the potential for FISH signals to merge in tandem repeats or as aggregates of double minutes and also the heterogeneity of results in individual tumor cells.

The cost of kit reagents in a single MLPA assay is less than $15. Although testing a tumor and normal brain DNA with two replicates each in three reactions to encompass probes for 79 genes increases the cost, testing multiple tumors with the same control DNA could be performed. Also, the number of genes to be screened could be reduced so that only 1 kit would be needed. Although high-level gene amplification results were obtained from the ultrasonic aspirations within a few days and the content of tumor DNA was plentiful, it should be noted that the FFPE assays demonstrated higher sensitivity in detection of low to mid-level gene amplifications. Therefore, ideally both types of specimens would be tested if detection of all levels of oncogene amplifications is desired. Multiplex detection of high-level amplifications using both types of samples strengthens confidence in the results at a relatively low cost and helps to negate concerns about tumor heterogeneity.

Although DNA from patients’ blood cells has been used as normal controls when searching for somatic genetic alterations in tumors, quantifying high-level amplified oncogenes pose a problem when using blood samples. Very low numbers of circulating tumor cells with high-level amplifications or their free DNA in the blood could bias analyses. Thus this study used normal brain DNA that was comparable to normal values expected with MLPA that were provided by the manufacturer. Pooled DNA from multiple normal donors should be considered for future studies. Based on this pilot study, automation of steps in the MLPA procedure and subsequent data analysis are future goals so that this type of assay can be streamlined, validated, and used clinically.

## Conclusions

In summary, multiplex detection of high-level amplifications among oncogenes was successful in ultrasonic surgical aspiration DNA obtained from malignant brain tumors when compared with FFPE from the same tumors. The results indicate that morphologic evaluation of ultrasonic surgical aspirations to confirm tumor cell content and retrieval of DNA would aid molecular testing of brain tumors for oncogene amplifications. Many of the oncogenes with copy number gains encode proteins that are potential therapeutic targets. Therefore rapid identification of high-level gene amplifications could stratify patients to clinical trials and treatment plans shortly after surgical resections of brain tumors. Although ultrasonic surgical aspiration specimens are less sensitive than FFPE in detecting low to mid-level amplifications, the bulk of the tumor is present in the aspirations, they are fresh and homogenously mixed, and high-level amplifications can be detected in them. Abundant tumor DNA harvested from cellular fluids could also be used for targeted sequencing of amplified oncogenes to detect activating mutations.

## Abbreviations

CE: Capillary electrophoresis; CGH: Comparative genomic hybridization; CN: Copy number; CUSA: Cavitronic ultrasonic surgical aspiration; DAP1: Diamino-phenylindole; EP1: Ependymoma; FFPE: Formalin-fixed: paraffin-embedded; FISH: Fluorescence in-situ hybridization; GBM: Glioblastoma multiforme; HPF: High power field; MLPA: Multiplex ligation-dependent probe amplification; LCM: Lung carcinoma metastasis; MHz: Mega Hertz; NB: Non-neoplastic (normal) brain; PCR: Polymerase chain reaction; SNP: Single nucleotide polymorphism.

## Competing interests

The authors declare that they have no competing interests.

## Authors' contributions

MEB, LNT, SSM, and JFL carried out the molecular genetic studies. MEB, LNT, and SP drafted the manuscript. SSM and MLN performed the FISH assays. MEB, SP, MLN and AN participated in the design of the study. SP, JFL, and AN participated in and coordinated specimen and clinical data retrieval and characterization. MEB conceived and coordinated the study. All authors read and approved the final manuscript. This study was conducted with approval from Institutional Review Board (IRB) of LSUHSC-S.
